# Living with migraine: A meta-synthesis of qualitative studies

**DOI:** 10.3389/fpsyg.2023.1129926

**Published:** 2023-03-28

**Authors:** Simone Battista, Arianna Lazzaretti, Ilaria Coppola, Luca Falsiroli Maistrello, Nadia Rania, Marco Testa

**Affiliations:** ^1^Department of Neurosciences, Rehabilitation, Ophthalmology, Genetics, Maternal and Child Health, University of Genova, Savona, Italy; ^2^Department of Clinical Sciences Lund, Orthopaedics, Lund University, Lund, Sweden; ^3^Department of Education Sciences, School of Social Sciences, University of Genova, Genova, Italy; ^4^Department of Physical Medicine and Rehabilitation, AULSS9 Scaligera, G. Fracastoro Hospital, San Bonifacio, Verona, Italy

**Keywords:** headache, quality of life, disease management, patient participation, decision making, rehabilitation

## Abstract

**Introduction:**

Migraine is one of the top ten causes of disability worldwide. However, migraine is still underrated in society, and the quality of care for this disease is scant. Qualitative research allows for giving voice to people and understanding the impact of their disease through their experience of it. This study aims at synthesising the state of the art of qualitative studies focused on how people with migraine experience their life and pathology.

**Methods:**

MEDLINE *via* PubMed, EMBASE, CINAHL, PsycINFO, and Cochrane Library were consulted up to November 2021 for qualitative studies. Studies to be eligible had to focus on adults (age > 18 years) with a diagnosis of primary episodic or chronic migraine following the International Classification of Headache. The quality of the study was analysed using the CASP (Critical Appraisal Skills Programme) tool. The synthesis was done through a thematic analysis. CERQual (Confidence in Evidence from Reviews of Qualitative research) approach was used to assess the confidence in retrieved evidence.

**Results:**

Ten studies were included, counting 262 people with migraine. Our synthesis produced four main themes. (1) “Negative impact of migraine symptoms on overall life” as migraine negatively impacts people's whole life. (2) “Impact of migraine on family, work and social relationship” as migraine reduces the possibility to focus at work and interact with people. (3) “Impact of migraine on emotional health” as people with migraine experience psychological distress. (4) “Coping strategies to deal with migraine” such as keep on living one's own life, no matter the symptoms.

**Conclusions:**

Migraine negatively impacts people's whole life, from private to social and work sphere. People with migraine feel stigmatised as others struggle with understanding their condition. Hence, it is necessary to improve awareness among society of this disabling condition, and the quality of care of these people, tackling this disease from a social and health-policy point of view.

## 1. Introduction

Migraine is a primary headache characterised by a throbbing pain on one side of the head, whose aetiology cannot be found in a specific structural alteration but in a combination of genetic and environmental factors (Burstein et al., 2015; Puledda et al., 2017). Migraine is the third most prevalent disorder worldwide and the second and third cause of disability and years of healthy life lost due to disability, respectively (Steiner et al., 2015, 2016, 2020). Moreover, it is one of the most common causes of absenteeism at work, and people with migraine experience a broad array of psychological distress due to their disease (Antonaci et al., 2011; Gandolfi et al., 2019; Donisi et al., 2020). Nevertheless, migraine is still underrated in society [World Health Organisation (WHO), 2011]. This underestimation of migraine disability is probably a result of a lack of education and knowledge of this disease among the general population and healthcare professionals [World Health Organisation (WHO), 2011; Guerrero et al., 2021; Pace et al., 2021].

The management of migraine is daunting as there is no definitive cure for this pathology, but symptoms-related management. People with migraine must learn how to coexist and cope with their disease. Recommendations for the treatment of acute migraine revolve around the importance of an early diagnosis and treatment, with the latter characterised by a personalised pharmacological intervention as first-line treatment (May and Schulte, 2016; Oskoui et al., 2019; Battista et al., 2021; Vanderpluym et al., 2021). Moreover, people with migraine should be educated on the lifestyle factors that can trigger or improve migraine attacks and the use of non-pharmacological treatments (e.g., muscular and relaxing techniques) (May and Schulte, 2016; Meyer et al., 2016; Falsiroli Maistrello et al., 2018; Garrigós-Pedrón et al., 2018). These treatments aim at reducing migraine frequency, duration and intensity. However, adherence to guidelines for the attack treatment of migraine is poor (Hepp et al., 2014; Olesen et al., 2022).

Considering the high impact of this disease, how underrated it is, and its difficult management, qualitative studies are needed to understand and give voice to people with migraine to understand their experience. By doing so, they allow for understanding people with different diseases, helping them in their therapeutic process, and improving their clinical management, influencing consultation behaviour and people's preferences (Peters et al., 2002; Noyes et al., 2018a). In 2002, Peters et al. stated that “few studies have been conducted on the patients' perspective on headache” (Peters et al., 2002). Since that moment, different qualitative studies have been published, leading to different systematic reviews. Nichols et al. analysed qualitative studies about the experience of different chronic headaches, including migraine (Nichols et al., 2017). However, migraine symptoms may overlap with other types of headaches, and chronic and episodic migraine might lead to different experiences worth exploring. Minen et al. conducted a meta-synthesis of qualitative studies on migraine management and patients' attitude to treatments and physicians (Minen et al., 2018). However, they did not take into account how people experience and live with this disease. In line with that, this study aims at filling the knowledge gap in the literature about people's perception of migraine (either episodic or chronic) and their implications on their life by synthetising qualitative studies on this topic.

## 2. Methods

A meta-synthesis is a systematic review and integration of findings from qualitative studies (Lachal et al., 2017). Meta-syntheses are concerned with understanding and describing key points, issues, and recurring themes within a research area of interest. Specifically, our meta-synthesis focuses on people's perception of a phenomenon (migraine) to offer different interpretations that might help the development of healthcare settings (Lachal et al., 2017). For this reason, the meta-synthesis approach suits the aim of this study, whose research question is: “How do people with migraine experience and manage their life?” The reporting of this meta-synthesis follows the Preferred Reporting Items for Systematic Reviews and Meta-Analyses statement (PRISMA) 2020 (Page et al., 2021).

### 2.1. Eligibility criteria

#### 2.1.1. Types of study

We included qualitative studies written in English and published in the last 21 years (2000–2021) that adopted different approaches (e.g., phenomenological analysis and grounded theory) and data collection methods (e.g., interviews and focus groups). Instead, we excluded studies in languages other than English that adopted quantitative designs such as systematic reviews, case reports, case series, and randomised-controlled trials (RCTs).

#### 2.1.2. Participants

We considered eligible all the studies that included adults (age > 18 years) with a diagnosis of primary episodic or chronic migraine following the criteria of the International Classification of Headache Disorders (ICHD), with or without aura,^1^ excluding people with a headache not classified as primary migraine headaches. We did not impose any restrictions on the sex and gender of participants.

#### 2.1.3. Types of evaluation

In this meta-synthesis, the focus is on people's experience of migraine. Thus, we included qualitative studies that focused on people with migraine. Instead, we excluded studies that concentrated only on caregivers or physicians.

### 2.2. Information sources

The research was conducted on MEDLINE *via* Pubmed, EMBASE, Cochrane Library, CINAHL, and PsycINFO. Since there is no consensus about which databases should be used for meta-synthesis, we adopted the recommendations from the “Cochrane Handbook for Systematic Reviews for Interventions” (Higgins et al., 2021). In their book, the Cochrane group suggested using MEDLINE *via* Pubmed, EMBASE, and Cochrane Library as the bare minimum requirement and adopted other sources based on the specific topic of the review. Therefore, we also adopted CINAHL and PsycINFO as they are preeminent databases for qualitative and psychological primary studies. We consulted these databases up to November 2021.

### 2.3. Search strategy

The search strategy adopted is the SPIDER tool used for qualitative evidence synthesis: Sample, Phenomenon of Interest, Design, Evaluation, and Research type (Cooke et al., 2012). The search strings used for all database is reported as Supplementary material 1. SB and AL conducted the search strategies with the help of a librarian from Lund University.

### 2.4. Selection process

Articles obtained from the research were uploaded to the Rayyan website after duplicate removal. Afterwards, two independent authors (AL and LFM) selected the studies applying the inclusion and exclusion criteria to titles and abstracts. In case of disagreement, a third author was consulted (SB). The full texts were read, and the final selection was decided through discussion by two authors (AL and SB). In addition to the inclusion and exclusion criteria, researchers evaluated the sample characteristics to include or not a study. The final purpose of this synthesis is to collect the experiences of a wide range of people with migraine, so if two studies had the same sample and similar settings, only one was included.

### 2.5. Data collection process

Two authors (AL and IC) independently extracted data from each study following the Cochrane indications (Noyes et al., 2018b) and using standardised Excel templates: author (year), title, country, setting, study design, objective, strengths and weaknesses, the total number of participants, sample characteristics, pathology of interest, frequency of migraine, and onset/years with migraine and disability rating scale. Then the two authors independently collected themes and subthemes from primary studies in a second Excel template. Disagreements in the data collection were resolved by either a consensus process or consultation with a third author (SB).

### 2.6. Methodological quality of the studies

Following Cochrane Qualitative and Implementation Group's recommendations, the studies were assessed for critical appraisal with the Critical Appraisal Skills Programme (CASP) tool by two authors independently (AL and IC) (Noyes et al., 2018b). CASP is the most common tool adopted for quality appraisal in health-related qualitative syntheses. The tool is made of ten questions that span from the use of appropriate methodology to the value of the results. Researchers can answer “yes”, “no”, or “can't tell” to each question. Each question has “comments” boxes to report why certain answers were given, and it is accompanied by suggested “hints” that help the researchers to reason upon the correct answer.

### 2.7. Data synthesis

A data-driven thematic analysis was used to synthesise the data with a descriptive approach (Dixon-Woods et al., 2005). Thematic analysis is a flexible method that identifies main or recurring themes from the included studies, summarising them under thematic headings. Specifically, data synthesis was divided into two phases. In the first one, two authors (AL and IC) thoroughly read the primary studies identifying their themes and subthemes independently. Then, they selected only those themes and subthemes that answered our research question, synthetising them based on their core meaning. In the second one, they discussed together their summarised themes and subthemes to reach a final consensus. In case of disagreement in the second phase, a third author (SB) was consulted.

### 2.8. Certainty of evidence

The Confidence in Evidence from Reviews of Qualitative research (CERQual) approach was used to assess the certainty of findings as either high, moderate, low or very low: it included the methodological limitations, relevance, coherence, and adequacy of data (Lewin et al., 2015). The methodological limitations of included studies were the result of the assessment made by the CASP tool. The relevance was the extent to which the setting or the inclusion criteria from the primary studies supporting review findings applied to the context specified in the review question (Lewin et al., 2015). The coherence assessed data consistency within and across all studies. The adequacy of data was an overall determination of the degree of richness and quantity of data supporting a review finding (Lewin et al., 2015).

## 3. Results

### 3.1. Study selection

**Figure 1:**
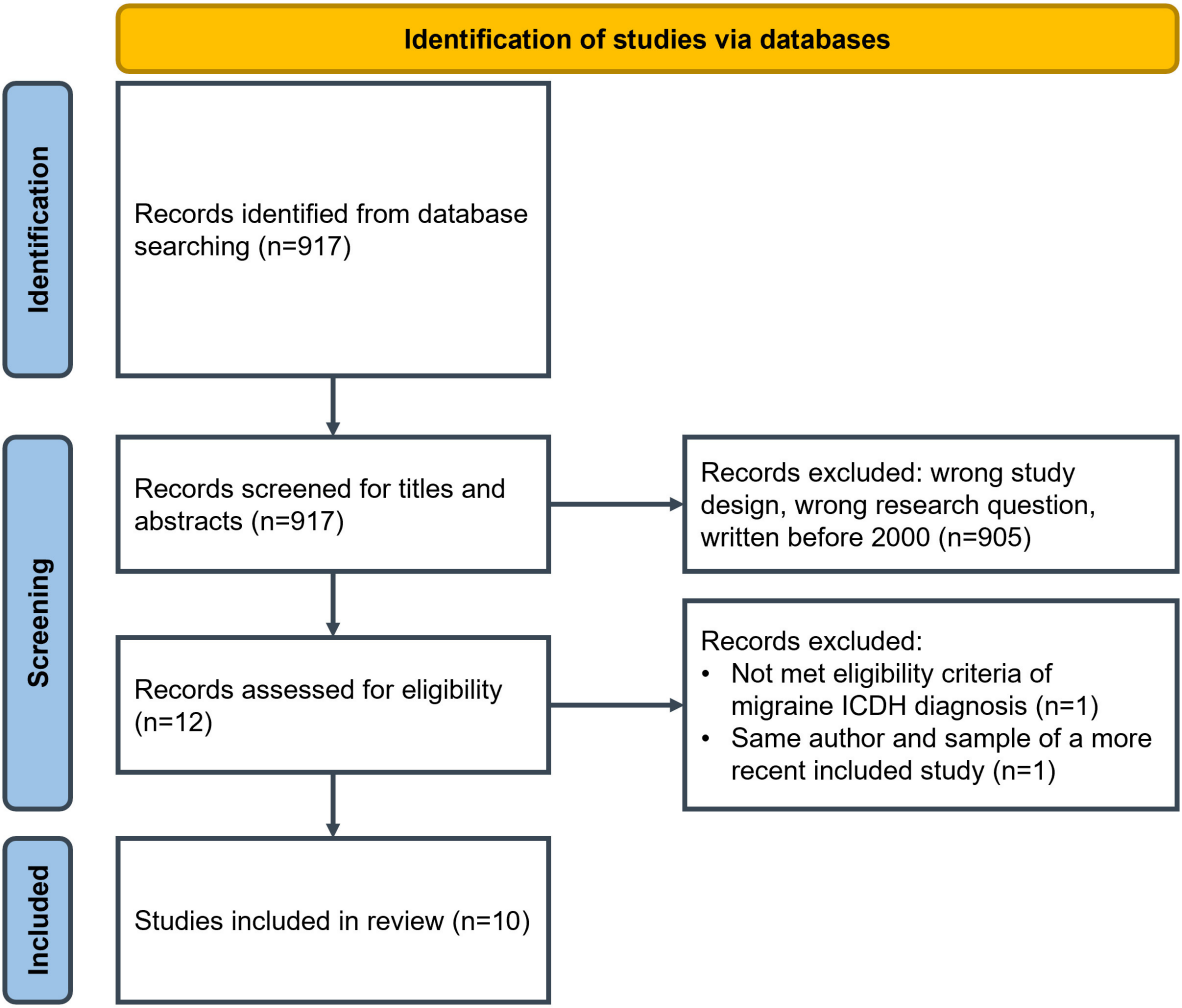
PRISMA 2020 flow diagram.

The research conducted on databases yielded 917 articles after the removal of duplicates. After the first screening selection of titles and abstracts, we excluded 905 studies. We read the full text of the remaining twelve articles. We excluded two studies as one did not declare a diagnosis of migraine following ICHD criteria (Leiper et al., 2006), and the other study (Moloney et al., 2004) presented the same sample (perimenopausal women) of a more recent study written by the same author included in this synthesis. Therefore, the final synthesis included ten articles (Cottrell et al., 2002; Ruiz De Velasco et al., 2003; Belam et al., 2005; Peters et al., 2005; Moloney et al., 2006; Ramsey, 2012; Rutberg and Öhrling, 2012; Palacios-Ceña et al., 2017; Scaratti et al., 2018; Estave et al., 2021) (Figure 1; PRISMA flow diagram).

### 3.2. Study characteristics

The ten studies included in the research counted 262 participants with a diagnosis of migraine headache (either episodic or chronic) according to ICHD criteria. Table 1 includes all study characteristics and the different themes and subthemes extracted by the authors of the articles.

**Table 1:** Summary of findings.

**References**	**Country**	**Study design and analysis**	**Sampling strategy**	**Population**	**Migraine and clinical characteristics**	**Themes and subthemes**
Estave et al. (2021), “Learning the full impact of migraine through patient voices: A qualitative study.”	United States of America	Semi-structured qualitative interviews analysed following a grounded theory	Participants were recruited from a pilot study and a RCT on the effect of a mindfulness-based stress reduction protocol in adults with migraine.	**Number:** 81 **Age:** Average 45–46 year (y) **Sex:** 90% female (F) **Ethnicity:** Caucasian **Pathology:** Migraine	•**Migraine onset:** Not available **(**N.A.) •**Years with migraine:** Pilot study and RCT: 26 •**Days with migraine (month):** •Pilot study: 4.2 RCT: 7.45 •**Frequency migraine attacks:** N.A. •**Days with use of symptomatic medication:** N.A. •**MIDAS** (Migraine Disability Assessment) - 1 months: Pilot study: 12.5 RCT: 13.7/10.0 •**HIT – 6** (Headache Impact Test 6): Pilot study and RCT: 63.0 •**Beck Depression Inventory, second edition (BDI-II):** N.A. •**State-Trait Anxiety Inventory (STAI):** N.A.	1. **Global negative impact on overall life:** (a) controls life; (b) makes life difficult; (c) causes disability during attacks; (d) lack of control over migraine attacks; (e) attempts to push through despite migraine. 2. **Migraine impact on emotional health:** (a) isolation; (b) anxiety; (c) frustration/anger; (d) guilt; (e) mood changes/irritability; (f) depression/hopelessness. 3. **Migraine impact on cognitive function:** (a) concentration difficulties, (b) communication challenges. 4. **Migraine impact on specific domains of life with resulting reactions:** (a) work/career: guilt, change of job status, presenteeism, financial impact, school impact; (b) family life: frustration, guilt, disrupted time; (c) social life: irritability, altered plans, communication. 5. **Fear and avoidance:** (a) pain catastrophising, (b) anticipatory anxiety, (c) avoidance behaviour. 6. **Stigma surrounding migraine:** (a) externalised stigma, (b) internalised stigma.
Palacios-Ceña et al. (2017), “Living with chronic migraine: qualitative study on female patients' perspectives from a specialised headache clinic in Spain.”	Spain	In-depth unstructured and semi-structured interviews and patients' drawings analysed following a phenomenological approach.	Patients were recruited at their first visit to the headache clinic at the Hospital Clìnico San Carlos (Madrid) neurology department. Sampling continued until redundant information from data analysis was achieved.	**Number:** 20 **Age:** mean age ± standard deviation (SD) 38.65 ± 13.85 **Sex:** 100% F **Ethnicity:** Caucasian **Pathology**: Chronic migraine	•**Migraine onset**: N.A. •**Years with migraine**: 20.2 (SD 13,23) •**Days with migraine** (month): 12.85 (SD 6.03) •**Frequency migraine attacks (month)**: 24.6 (SD 4.7) •**Days with use of symptomatic medication** (month): 14.1 (SD 8.91). •**MIDAS**: N.A. •**HIT – 6**: N.A. •**BDI-II:** five patients had mild depression and three had moderate depression. •**STAI:** fourteen patients with some degree of anxiety (moderate to severe).	**1. The shame of suffering from an invisible condition;** 2. **Treatment: between need, scepticism and fear;** 3. **Looking for physicians' support and sincerity and fighting misconceptions;** 4. **Limiting the impact on daily life through self-control;** 5. **Family and work: between understanding and disbelief**.
Rutberg and Öhrling (2012), “Migraine – more than a headache: women's experiences of living with migraine.”	Sweden	In-depth interviews and drawings following a Hermeneutic phenomenological method.	Letters describing the purpose of the study were sent to all 24 members of Swedish Migraine Association. Those who showed interest were contacted by phone, and they all gave written informed consent.	**Number:** 10 **Age** (range): between 37 and 69 **Sex:** 100% F **Ethnicity:** Caucasian **Pathology:** Migraine	•**Migraine onset (age):** eight women migraine started in their late teens or their early twenties. Two women migraine started in menopause. •**Years with migraine:** N.A. •**Days with migraine (month):** N.A. •**Frequency migraine attacks (number):** One-two attack(s) per year for two women, one-four attacks per month for six women and 10-20 attacks per month for two women •**Days with use of symptomatic medication (month):** N.A. •**MIDAS**: N.A. •**HIT – 6:** N.A. •**BDI-II:** N.A. •**STAI:** fourteen patients with some degree of anxiety (moderate to severe).	1. **Being besieged by an attack:** (a) being temporarily incapacitated; (b) feeling involuntarily isolated from life. 2. **Struggling in a life characterised by uncertainty:** (a) being in a state of constant readiness; (b) worrying about the use of medication. 3. **Living with an invisible disorder:** (a) living with the fear of not being believed; (b) struggling to avoid being doubted.
Ramsey (2012), “Living with migraine headache: a phenomenological study of women's experiences.”	United States of America	Hermeneutic Phenomenological inquiry and storey theory with interviews.	Women who held an account at a mid-Atlantic university received an illustrative e-mail. More than 100 women wanted to participate, but the researcher contacted the first 12 who supplied a phone number. The authors decided that redundancy was evident in the eight participant storey.	**Number:** eight **Age:** Average 35,9 y **Sex:** 100% F **Ethnicity:** Caucasian **Pathology:** Migraine	•**Migraine onset (age):** average 20,5 y •**Years with migraine:** N.A. •**Days with migraine (month):** N.A. •**Frequency migraine attacks (number):** N.A. •**Days with use of symptomatic medication (month):** N.A. •**MIDAS:** N.A. •**HIT – 6:** N.A. •**BDI-II:** N.A. •**STAI:** N.A.	**1. Recalling the significant experience that reshaped life; 2. Experiencing self as vulnerable, with unmet expectations, unfulfilled relationship, and regrets; 3. Being overcome by unrelenting, torturous pain magnified by intrusion from the outside world; 4. Pushing through to hold self together to do what needs to be done despite tortuous pain; 5. Surrendering to the compelling call to focus on self in order to relieve the torturous pain; 6. Making the most of pain-free time to get on with life and navigate the aftermath of the headache experience; 7. Being on guard against an unpredictable attack and yet hopeful that it is possible to outsmart the next attack**.
Peters et al. (2005), “The patients' perceptions of migraine and chronic daily headache: a qualitative study.”	United Kingdom	Semi-structured interviews analysed following grounded theory methodology.	Participants were recruited in Surrey (UK) by personal contact, posters in two local supermarkets and letters to 20 members of the Migraine Action Association.	**Number:** 13 **Age:** average 42,7 y **Sex: nine** male (M) and four female. **Ethnicity:** Caucasian **Pathology:** Migraine, five participants also had chronic daily headache (CDH) with >15 attacks per month and nine had tension-type headache (TTH).	•**Migraine onset (age):** N.A. •**Years with migraine:** N.A. •**Days with migraine (month):** N.A. •**Frequency migraine attacks (number): five** participants had >15 attacks per month. •**Days with use of symptomatic medication (month):** N.A. •**MIDAS:** four participants minimal; one mild; six moderate (three with migraine and three with CDH), two severe disability (CDH). •**HIT – 6:** N.A. •**BDI-II:** N.A. •**STAI:** N.A.	1. **Headaches:** (a) pain and other symptoms; (b) differentiating between different types of headache; (c) perceptions of headaches as barriers and facilitators to care. 2. **Headache impact**. 3. **Headache as a health issue**.
Scaratti et al. (2018), “A qualitative study on patients with chronic migraine with medication overuse headache: comparing frequent and non-frequent relapsers.”	Italy	In-person interviews analysed following thematic analysis and a narrative approach.	Participants were consecutively recruited during structured withdrawal treatments at the Headaches Centre of the Neurological Institute C. Besta in Milan between November 2015 and June 2016. Inclusion criteria: >18 years old, diagnosis of chronic migraine and medication overuse.	**Number:** 16 **Age:** mean age 53 y **Sex:** 13 F, 3 M **Ethnicity:** Caucasian **Pathology:** Chronic migraine and medication overuse headache (MOH). Seven participants were classified as frequent re relapsers (FRs) and nine as non-frequent relapsers (NFRs). Patients had both psychiatric (depression or anxiety) and physical comorbidities.	•**Migraine onset (age):** N.A. •**Years with migraine:** FRs 18 years; NFRs 13 years. •**Days with migraine (month):** average 21-22 •**Frequency migraine attacks (number):** N.A. •**Days with use of symptomatic medication (month):** N.A. •**MIDAS:** N.A. •**HIT – 6:** N.A. •**BDI-II:** N.A. •**STAI:** N.A.	1. **Disclosing or concealing headache and the dilemma of isolation;** 2. **Medication addiction;** 3. **Anxiety;** 4. **Use of non-pharmacological therapies**.
Cottrell et al. (2002), “Perceptions and needs of patients with migraine: a focus group study.”	United States of America	Focus groups analysed following thematic analysis.	Names of potential participants were obtained from a list of people recruited for a separate headache study conducted by two of the authors; telephone screening.	**Number:** 24 **Age:** range between 25 and 49 y **Sex:** 100% F **Ethnicity:** Caucasian **Pathology:** Migraine, two participants had also occasional tension type headache (TTH).	•**Migraine onset (age):** N.A. •**Years with migraine:** Authors included patients who had experienced migraine for at least six months. •**Days with migraine (month):** one-two. •**Frequency migraine attacks (number):** two third of sample had one to three per month. •**Days with use of symptomatic medication (month):** N.A. •**MIDAS:** N.A. •**HIT – 6:** N.A. •**BDI-II:** N.A. •**STAI:** N.A.	1. **Effect on social functioning;** 2. **Effect on family functioning;** 3. **Effect on work;** 4. **Effect on relationships;** 5. **Issues related to physician care;** 6. **Problems with insurance and drug companies**.
Moloney et al. (2006), “The experiences of midlife women with migraines.”	United States of America	Data were collected in two consecutive multi-method studies: the first one used qualitative interviews, focus group, paper-and-pencil questionnaire (HHQ, Migraine-Specific QoL, SF-36) and six-month daily diaries. The second study was internet-based with both in-person and phone interviews, similar quantitative questionnaires and virtual focus groups (online discussion boards). The interpretative hermeneutic approach was used for analysis.	Ten participants in the first study were recruited from a health maintenance organisation. Forty-three participants in the second study were recruited from a university setting, the local community and the internet.	**Number:** 53 **Age:** range between 40 and 55. **Sex:** 100% F (perimenopausal women). **Ethnicity:** 44 Caucasian, eight African American, one English Indian. **Pathology:** Migraine.	•**Migraine onset (age):** N.A. •**Years with migraine:** N.A. •**Days with migraine (month):** N.A. •**Frequency migraine attacks (number):** two/three •**Days with use of symptomatic medication (month):** N.A. •**MIDAS:** N.A. •**HIT – 6:** N.A. •**BDI-II:** N.A. •**STAI:** N.A.	1. **Shifting headache patterns:** (a) headaches patterns; (b) looking for an answer; 2. **Predicting, preventing, and controlling headaches:** (a) is this a migraine or something else?; (b) identifying triggers; (c) course of headache: the lurking migraine; (d) medications; (e) I might try…: self-care interventions; 3. **Keeping on the move:** (a) working through headache; (b) desperation; (c) keeping my arsenal of medicine; (d) having a dirty secret.
Belam et al. (2005), “A qualitative study of migraine involving patient researchers.”	United Kingdom	Qualitative interviews analysed following a grounded theory.	Patient researchers were recruited from a local intermediate care headache clinic, advertised through the local press, word of mouth and an organisation for people with migraine. Study participants were recruited from a local headache clinic.	**Number:** eight **Age:** average 47,6 **Sex:** six F and 2 M **Ethnicity:** Caucasian **Pathology:** Migraine	•**Migraine onset (age):** N.A. •**Years with migraine:** N.A. •**Days with migraine (month):** N.A. •**Frequency migraine attacks (number):** two/three •**Days with use of symptomatic medication (month):** N.A. •**MIDAS:** N.A. •**HIT – 6:** average 70,5 (all results were over 56 that means substantial impact) •**BDI-II:** N.A. •**STAI:** N.A.	1. **Impact on life (everyone is different):** (a) physical and psychological impact; (b) impact on family and social life; (c) impact on career. 2. **Making sense of the problem; Putting up with it;** 3. **Doing something about it:** (a) self-help; (b) professional help.
Ruiz De Velasco et al. (2003), “Quality of life in migraine patients: a qualitative study.”	Spain	Six focus groups and nine personal interviews. The method used for the analysis was described by Krueger: the researcher offers brief descriptions based on direct data followed by an illustrative example.	Participants were divided in six groups: in the first, second and third groups, patients were recruited from the Department of Neurology of Hospital de Galdakao, Spain. In the fourth group, participants were selected by pharmacists; the fifth group included healthcare professionals (nurses and physicians); the last group included relatives of patients with migraine.	**Number:** 41 (29 migraine suffers) **Age (average):** first group: 35, 43; second group: 37, 66; third group: 34, 13; fourth group: 48, 5. **Sex:** 30 F overall (27 F and 2 M migraine suffers). **Ethnicity:** Caucasian **Pathology:** Migraine with or without aura.	•**Migraine onset (age):** N.A. •**Years with migraine:** N.A. •**Days with migraine (month):** N.A. •**Frequency migraine attacks (number):** first group: 3,4 (range 2-6); second group: 5,3 (range 2-11); third group: 6,7 (range 2-12); fourth group 3 (range 2-9). •**Days with use of symptomatic medication (month):** N.A. First group used prophylaxis (nadolol 70%, amitriptyline 20%, flunarizine 10%). •**MIDAS:** N.A. •**HIT – 6:** N.A. •**BDI-II:** N.A. •**STAI:** N.A.	1. **Symptomatic aspects; Social aspects:** (a) work and studies; (b) family relationships; (c) social relationships; 2. **Emotional aspects**.

### 3.3. Methodological quality of the studies

The overall evaluations of CASP are collected in Table 2. The single answers with respective explanations for all the studies are reported in Table 3.

**Table 2:** Evaluations of methodological quality of the studies—CASP checklist.

**Question**	**Yes (Number of studies)**	**Can't tell (Number of studies)**	**No (Number of studies)**
1. Was there a clear statement of the aims of the research?	10	0	0
2. Is a qualitative methodology appropriate?	10	0	0
3. Was the research design appropriate to address the aims of the research?	6	4	0
4. Was the recruitment strategy appropriate to the aims of the research?	8	1	1
5. Was the data collected in a way that addressed the research issue?	7	3	0
6. Has the relationship between researchers and participants been adequately considered?	5	5	0
7. Have ethical issues been taken into consideration?	4	6	0
8. Was the data analysis sufficiently rigorous?	10	0	0
9. Is there a clear statement of findings?	10	0	0
10. How valuable is the research?	10	0	0

**Table 3:** Answers explanations of CASP.

**References**	**1. Was there a clear statement of the aims of the research?**	**2. Is a qualitative methodology appropriate?**	**3. Was the research design appropriate to address the aims of the research?**	**4. Was the recruitment strategy appropriate to the aims of the research?**	**5. Was the data collected in a way that addressed the research issue?**	**6. Has the relationship between researchers and participants been adequately considered?**	**7. Have ethical issues been taken into consideration?**	**8. Was the data analysis sufficiently rigorous?**	**9. Is there a clear statement of findings?**	**10. How valuable is the research?**
Estave et al. (2021), “Learning the full impact of migraine through patient voices: A qualitative study.”	Yes	Yes	Can't tell (it does not explain why they use grounded theory, even if the results seem coherent with the approach)	Can't tell (participants take part in two RCTs and the recruitment strategy is explained in another paper)	Can't tell (it does not explain why they use grounded theory, even if the results seem coherent with the approach)	Yes	Yes	Yes	Yes	The authors specific in the paragraph “strengths and limitations” the contribution of their study to the existing knowledge and its limitations, such as selection bias and the difficulty of transferring the findings to other populations.
Palacios-Ceña et al. (2017), “Living with chronic migraine: a qualitative study on female patients' perspectives from a specialised headache clinic in Spain.”	Yes	Yes	Yes	Yes	Yes	Yes	Yes	Yes	Yes	The authors discussed the strengths and limitations of the study in the paragraph “Discussion.” A limitation is the low generalisability due to the women sample. The authors discuss the contributions to existing knowledge explaining that their study is the first to treat chronic migraine and compare their findings with ones in current literature.
Rutberg and Öhrling (2012), “Migraine – more than a headache: women's experiences of living with migraine.”	Yes	Yes	Yes	Yes	Yes	Can't tell (because the considerations explained in the paragraph “*Justification of the study”* are not enough to understand the relationship between researchers and participants)	Yes	Yes	Yes	The authors declare the strengths and limitations of the study in the paragraph “Methodological considerations.” A limitation is the sample of only women that do not allow for generalising the data to other genders. The authors compare their findings to the current literature in the paragraph “Discussion.”
Ramsey (2012), “Living with migraine headache: a phenomenological study of women's experiences.”	Yes	Yes	Yes	No (Because the paragraph “*Data collection”* did not explain why they contacted only the first 12 volunteers, which does not justify their relevance in responding to the research question).	Yes	Yes	Can't tell (There is no code or date of approval).	Yes	Yes	The authors discuss the generalisability of their findings and the implications of practise in the paragraph “Implications for holistic nursing practise.”
Peters et al. (2005), “The patients' perceptions of migraine and chronic daily headache: a qualitative study.”	Yes	Yes	Can't tell (it is explained in another paper and the authors do not explain why they use this research design to answer the research question)	Yes	Yes	Can't tell (problem on reporting)	Can't tell (Ethical approval was obtained from the University of Surrey Ethics Committee, but there is no code)	Yes	Yes	In the paragraph “Discussion” is presented the information this study adds to current literature and which are the further step to investigate. The author discuss the limitations to the generalisability of findings due to the small sample size and the nature of the qualitative analysis.
Scaratti et al. (2018), “A qualitative study on patients with chronic migraine with medication overuse headache: comparing frequent and non-frequent relapsers.”	Yes	Yes	Yes	Yes	Yes	Yes	Can't tell (the ethical committee of the Institute approved the study, but there is neither a code nor the date of approval)	Yes	Yes	In the paragraph “Discussion” the authors explained the value of their approach that was “data-driven” and underlined the limitations such as the not precise definition of FR and the low applicability due to the
										limited number of participants. The authors explain in the paragraph “Conclusion” the implications for the clinical practise such as considering some relevant psychological aspects of patients.
Cottrell et al. (2002), “Perceptions and needs of patients with migraine: a focus group study.”	Yes	Yes	Can't tell (the authors do not explain why they use this research design to answer the research question).	Yes	Can't tell (it is not specified why they chose the focus group).	Can't tell (the relationship between researchers and participants is not reported and explained).	Can't tell (there is neither a code nor a date of approval)	Yes	Yes	The authors underline the limitations of the study in the paragraph “Discussion” such as the small sample size and the characteristics of participants that are not generalisable. Authors compare their findings to the current literature and suggest implications for practise lie in need for more general information about migraines and their management.
Moloney et al. (2006), “The experiences of midlife women with migraines.”	Yes	Yes	Can't tell (the authors don't specify why they use the hermeneutic approach)	Yes	Can't tell (the research issue is not adequately explained)	Can't tell (the relationship between researchers and participants is not reported and explained).	Can't tell (there is neither a code nor date of approval)	Yes	Yes	The authors discuss their findings compared to current literature in the paragraph “Discussion.” A paragraph is dedicate to “Implications for research, practise and education.”
Belam et al. (2005), “A qualitative study of migraine involving patient researchers.”	Yes	Yes	Yes	Yes	Yes	Yes	Yes	Yes	Yes	The authors accepted a lack of rigor because the perspective is more influenced by action research, but underlined the different insights into the investigations that resulted in a practical approach. The authors discussed strengths and weaknesses in the paragraph “Strengths and limitations of this study.”
Ruiz De Velasco et al. (2003), “Quality of life in migraine patients: a qualitative study.”	Yes	Yes	Yes	Yes	Yes	Can't tell (the relationship between researchers and participants is not adequately reported and explained)	Can't tell (there is neither a code nor date of approval)	Yes	Yes	The authors explain strengths and limitations in the paragraph “Discussion” and discuss the contribution to existing knowledge: the perspective of self-medicated patients, family relatives and healthcare professionals.

### 3.4. Results of the synthesis

The synthesis produced four main themes, as shown in Table 4. Every main theme was examined in some subthemes to explain more clearly the various life aspects affected by migraine.

**Table 4:** Final themes and subthemes.

**Themes**	**Subthemes**
1. Negative impact of migraine symptoms on overall life	- Everything is about pain - Disabling symptoms and physical impact - Migraine involves day-to-day life - Inability to carry out activities with pleasure (want to but not able to)
2. Impact of migraine on family, social and work relationships	- Migraine affects cognitive function (loss of concentration/memory) at work to the point where it may result in a need to change or lose the job - People with migraine are often not understood by their bosses or friends (it is not even considered serious) - Migraine affects the ability to take care of children - Negative impact on the relationship with partner (including sexual relation) - Migraine affects social life (leisure activities, sports, holidays)
3. Impact of migraine on emotional health	- Migraine involves psychological distress (avoidance behaviour, anticipatory anxiety, depression) - Migraine affects intrapersonal emotions (frustration, desperation, irritability, mood changes and hopelessness) - Consequences of social and family aspects on emotional health (isolated, guilty)
4. Coping strategies to deal with migraine	- Self-efficacy before and during an attack (focus on self) - Take advantage of pain-free time - Share experiences - Balance the demands of life

#### 3.4.1. Theme 1: Negative impact of migraine symptoms on overall life

The first theme was present in most studies (Ruiz De Velasco et al., 2003; Belam et al., 2005; Peters et al., 2005; Ramsey, 2012; Rutberg and Öhrling, 2012; Palacios-Ceña et al., 2017; Estave et al., 2021). It deals with how migraine affects patients' lives through physical symptoms, pain and the consequent inability to function at their best. This was the first theme that came to light because it explained how migraine negatively affected the lives of people with it and represented the underlying cause of the most negative experiences that emerged in the following subthemes.

##### 3.4.1.1. Subtheme 1A: Everything is about pain

The participants described the pain as routine using a vivid range of metaphors to explain how impactful migraine was on them:

“A freight train coming through”, “A storm entering my head”, “As if my head would explode” (Ramsey, 2012).

“It's like somebody's put a knife through my head. The pain is so intense that for several seconds I don't even open my eyes, in the hope that I'm just dreaming about it” (Peters et al., 2005).

##### 3.4.1.2. Subtheme 1B: Disabling symptoms and physical impact

Participants also experienced physical and disabling symptoms such as nausea, vomiting, and visual or auditory impairment (aura). Aura did not affect all people with migraine, but it was considered one of the most disabling symptoms.

“Hearing that all day would kill me”, “A stereo that someone just keeps turning the volume up in my head”, “As echoing through my head”, “As fingernails on a chalkboard” (Ramsey, 2012).

“And your eyes begin to close because your whole body hurts and you feel pain when there is any kind of noise, light, anything at all” (Ruiz De Velasco et al., 2003).

##### 3.4.1.3. Subtheme 1C: Migraine involves day-to-day life

People with migraine reported that their disease affected their life and hindered their ability to live it.

“I am losing a day of my life”, “Attacks make doing day-to-day things a lot more difficult. […] It makes day-to-day living harder” (Estave et al., 2021).

“You lose your life for a moment” (Rutberg and Öhrling, 2012).

##### 3.4.1.4. Subtheme 1D: Inability to carry out activities with pleasure (want to but not able to)

Migraine symptoms also cause a loss of pleasure in daily activities.

“I have to stop doing things that I like to do, and I can't enjoy things I like to do”, “I never felt real joy because of always having this in the back of my mind” (Estave et al., 2021).

#### 3.4.2. Theme 2: Impact of migraine on family, social, and work relationships

The second theme focused on how migraine affects people's relationships (Cottrell et al., 2002; Ruiz De Velasco et al., 2003; Belam et al., 2005; Peters et al., 2005; Ramsey, 2012; Rutberg and Öhrling, 2012; Palacios-Ceña et al., 2017; Scaratti et al., 2018; Estave et al., 2021). They explained how others considered them and how difficult it is to get along with social life. Participants voiced a problematic concept of not being understood by others, especially in the workplace where there could be consequences on their career up until the loss of their job. This problem sometimes emerged among friends and family. People with migraine perceived a certain sense of disbelief from others while they explained their situation as it is an “invisible condition”. The theme of failing to take care of children was recurrent in the studies (Cottrell et al., 2002; Belam et al., 2005; Ramsey, 2012; Estave et al., 2021). Moreover, a few participants expressed the negative impact on sexual relations voicing a common discomfort that was not often mentioned because of shame.

##### 3.4.2.1. Subtheme 2A: Migraine affects cognitive function (loss of concentration/memory) at work and people feel they have to change their job or they even lose it

The participants complained about the effect of migraine on their work. This conception was recurring among the studies because migraine attacks also involved cognitive functions, and participants underlined the consequences of work.

“I've been fired from a job before because of my migraine attacks” (Estave et al., 2021).

“When I've got a migraine, I know that I can't give 100%, and that bothers me” (Ramsey, 2012).

“I try to look productive, but I'm only doing half” (Cottrell et al., 2002).

“It affects my career choice” (Belam et al., 2005).

“It's hard to concentrate”; “It affects memory” (Rutberg and Öhrling, 2012).

“There is this fear that if I get (a migraine) I'm gonna have to dive off (work), and I won't be able to fulfil duties” (Peters et al., 2005).

In most studies, participants voiced the theme of not being understood at work and its consequences on their work experiences.

“They thought it was a joke because nobody takes it seriously and nobody knows what migraine is”, “They've never had it they just think it's a headache, and it's not just a headache” (Estave et al., 2021).

“My workmate told my bosses that if I had a headache, I should take a pill and that it was no excuse not to go to work” (Palacios-Ceña et al., 2017).

##### 3.4.2.2. Subtheme 2B: Migraine affects the ability to take care of children

Migraine often made childcare difficult, according to participants, who expressed it this way:

“I feel like I can't take care of him (18-month-old)” (Estave et al., 2021).

“It's very difficult to think that there are times when you can't take care of your child” (Ramsey, 2012).

“Mummy just can't deal with them [games] or do any housework or do anything” (Peters et al., 2005).

“I'm not the mom I wanted to be” (Cottrell et al., 2002).

“My son is only 11 and he has never known me any different” (Belam et al., 2005).

##### 3.4.2.3. Subtheme 2C: Negative impact on the relationship with partner (including sexual relation)

The consequences of migraine attacks were also reported in the relationship with the partner, as the participants explained:

“It affects my husband because it puts more on him when I have one” (Estave et al., 2021).

“It's changing my life even in our sexual relations because since I began to have this pain, I haven't felt any kind of sexual arousal” (Ruiz De Velasco et al., 2003).

##### 3.4.2.4. Subtheme 2D: Migraine affects social life (leisure activities, sports, holidays)

Participants' experience of migraine also involved social life.

“You can't lead a normal life, you can't go out dancing, to dinner, to the cinema. It changes the way you live.”, “It limits the time I can spend with my friends and even the desire to do sport” (Palacios-Ceña et al., 2017).

“Social life is affected a lot…I no longer have any relationship with them (friends)… the others, after a while, got tired of me” (Scaratti et al., 2018).

Moreover, participants reported their friends and acquaintances do not completely understand their situation. They struggle with legitimising it.

“I think people look like—yeah, right, everybody has headaches. They're not that bad, just get a grip and keep going” (Cottrell et al., 2002).

“The others don't understand because it is a sharp pain, and if you haven't experienced it, you can't imagine what it's like” (Ruiz De Velasco et al., 2003).

#### 3.4.3. Theme 3: Impact of migraine on emotional health

The third theme dealt with emotional features that followed migraine and affected participants' lives even from a psychological aspect. Migraine involves psychological distress (avoidance behaviour, anticipatory anxiety, and depression). Psychological distress was common among participants, who suffered a lot and often presented themselves as overwhelmed by this condition (Ruiz De Velasco et al., 2003; Belam et al., 2005; Moloney et al., 2006; Ramsey, 2012; Rutberg and Öhrling, 2012; Palacios-Ceña et al., 2017; Scaratti et al., 2018; Estave et al., 2021).

##### 3.4.3.1. Subtheme 3A: Migraine involves intrapersonal emotions (frustration, desperation, irritability, mood changes, depression, anxiety, and hopelessness)

Participants expressed their emotions, such as frustration and desperation, with condition that was difficult to explain and face. Emotions such as irritability and mood changes also affected the social relation triggering a vicious circle of discomfort.

“I'm more irritable and don't want to be around a lot of people” (Estave et al., 2021).

“Desperation is definitely part of the day” (Moloney et al., 2006).

“You are always in a bad mood, and besides” (Ruiz De Velasco et al., 2003).

“I get in such a bad mood that I can't stand anyone, you're irritable, you do not anyone talk to you, no-one to tell you anything” (Palacios-Ceña et al., 2017).

Among the different feelings, depression and anxiety were the most reported ones:

“[Attacks] cause a lot of anxiety because I don't know when I'm going to have one and I'm fearful. And when I have one, I'm fearful it's not going away” (Estave et al., 2021).

“I feel a little depressed. […] I can't react anymore, I'm tired of my headache” (Scaratti et al., 2018).

##### 3.4.3.2. Subtheme 3B: Consequences of social and family aspects on emotional health (isolated, guilty)

Participants of Estave's study explained that physical and psychological symptoms led to feelings of isolation and guilty about time away from social engagement and family duties:

“I'm sorry it affects me because it takes me away from my family, my kids”, “My daughters, my husband and everybody … they just stopped including me in everything, so I felt like I was observing them live, but I wasn't really living” (Estave et al., 2021).

Participants of the studies by Palacios-Ceña et al. (2017) and Scaratti et al. (2018) explained the feeling of isolation:

“I am isolated from almost all of the people I know, except from my family of origin and from some friends…but I no longer have any relationship with them…the others, after a while, got tired of me” (Scaratti et al., 2018).

“It cuts you off from being with others; it separates you from everyone else” (Palacios-Ceña et al., 2017).

#### 3.4.4. Theme 4: Coping strategies to deal with migraine

The last theme underlined the coping strategies that participants adopt to deal with their migraine. Participants voiced concern about the implications of migraine on every aspect of life, and, in most cases, it was hard to take on. However, they shared the strategies they adopted against the disability caused by attacks to cope with migraine.

##### 3.4.4.1. Subtheme 4A: Self-efficacy as a support to manage migraine

Participants expressed their willingness not to be overwhelmed by pain. Therefore, they lived trying to go through the attack, managing it (Palacios-Ceña et al., 2017). They explained their will to keep on doing their activities, no matter the symptoms, to meet their expectations in a social or work context (Ramsey, 2012). However, they also showed to be aware of when taking care of themselves (Ramsey, 2012). Belam et al., in their study, talked about how people adopted self-help strategies to cope with attacks and look for remedies (Belam et al., 2005). The participants in Moloney et al. study added that it was essential to focus on causes and triggers to increase prediction and control (Moloney et al., 2006).

“You try not to let it affect you, to control everything, to deal with it, to be conscious of everything that might cause pain.” “I try to tolerate the pain as much as I can” (Palacios-Ceña et al., 2017).

“ […] you just have to go on through it” (Ramsey, 2012).

##### 3.4.4.2. Subtheme 4B: Take advantage of pain-free time

Another strategy voiced by participants was using time devoid of pain to engage in activities like exercise and stress reduction to prevent other attacks and reduce the frequency.

“The good things are certainly that you don't have headache, but sometimes during the inactive phase you're actually getting over another one, and so you're trying to recoup, and sometimes redo things that you have done halfway […]. I try to take those inactive times to really enjoy life” (Ramsey, 2012).

##### 3.4.4.3. Subtheme 4C: Share experiences

Participants voiced their need to share experiences, talk to others and explore meaning as they want to understand their condition and adjust it in the context of their lives.

“It was very helpful to be able to talk to and listen to other people who suffer from migraine”, “When you realise that other members of the family have migraine, you feel the battle is over—you understand why you get them” (Belam et al., 2005).

##### 3.4.4.4. Subtheme 4D: Balance the demands of life

Living with migraine was a constantly evolving process that required constant attention and vigilance. This process included the ability to balance the demands of life.

“You learn to live with it, and you do not know what life would be without it, but it is like permanently wearing a backpack, which is though, you must always consider the possibility of not being able to do things” (Rutberg and Öhrling, 2012).

Participants voiced that they lived in a constant state of readiness to avoid triggers and control the attack. They described migraine with this metaphor:

“It's though that I am forced to live with somebody who always interrupts and decides what I should or should not do” (Rutberg and Öhrling, 2012).

### 3.5. Certainty of evidence

Table 5 reports the certainty of quality evidence (CerQual approach). None of the study findings was evaluated to be higher certainty because of weaknesses in relevance and minor methodology limitations of included studies. All the study findings were assessed as moderate confidence, which meant a good level of certainty because of minor concerns regarding the coherence and adequacy of data within and across all studies included.

**Table 5:** Certainty of evidence (CerQual).

**Review finding**	**Studies contributing to the review finding**	**Assessment of methodological limitations**	**Assessment of relevance**	**Assessment of coherence**	**Assessment of adequacy of data**	**Overall CerQual assessment of confidence**	**Explanation of judgement**
Negative impact of migraine symptoms on overall life	Ruiz De Velasco et al., 2003; Belam et al., 2005; Peters et al., 2005; Ramsey, 2012; Rutberg and Öhrling, 2012; Palacios-Ceña et al., 2017; Estave et al., 2021	Minor methodological limitations (two studies with no limitations, one with minor limitations on research design, recruitment strategy and data collections, one study with moderate methodological limitations on recruitment strategy and the other studies have minor methodological limitations)	Substantial concerns about relevance (all the studies included only Caucasian people)	Minor concerns about coherence (data reasonably consistent within and across all studies)	Minor concerns about adequacy (seven studies that offered together moderately rich data overall)	Moderate confidence	This finding was graded as moderate confidence because of minor concerns regarding methodological limitations, coherence and adequacy; though substantial concerns about relevance.
Impact of migraine on family, work and social relationships	Cottrell et al., 2002; Ruiz De Velasco et al., 2003; Belam et al., 2005; Peters et al., 2005; Ramsey, 2012; Rutberg and Öhrling, 2012; Palacios-Ceña et al., 2017; Scaratti et al., 2018; Estave et al., 2021	Minor methodological limitations (two studies with no limitations, one study with concerns on research design and data collection, one study with concerns with research design, recruitment strategy and data collection, one with moderate concern on recruitment strategy and the other studies have minor methodological limitations)	Substantial concerns about relevance (all the studies included only Caucasian people)	Minor concerns about coherence (data reasonably consistent within and across all studies)	Minor concerns about adequacy (nine studies that offered together moderately rich data overall)	Moderate confidence	This finding was graded as moderate confidence because of minor concerns regarding methodological limitations, coherence and adequacy; though substantial concerns about relevance.
Impact of migraine on emotional health	Ruiz De Velasco et al., 2003; Belam et al., 2005; Moloney et al., 2006; Ramsey, 2012; Rutberg and Öhrling, 2012; Palacios-Ceña et al., 2017; Scaratti et al., 2018; Estave et al., 2021	Minor methodological limitations (two studies with no limitations, one study with concern on research design, recruitment strategy and data collection, one study with minor concern on research design and data collection, one study with moderate concern on recruitment strategy and the other studies have minor methodological limitations)	Substantial concerns about relevance (all the studies included only Caucasian people)	Minor concerns about coherence (data reasonably consistent within and across all studies)	Minor concerns about adequacy (eight studies that offered together moderately rich data overall)	Moderate confidence	This finding was graded as moderate confidence because of minor concerns regarding methodological limitations, coherence and adequacy; though substantial concerns about relevance.
Coping strategies to deal with migraine	Belam et al., 2005; Moloney et al., 2006; Ramsey, 2012; Rutberg and Öhrling, 2012; Palacios-Ceña et al., 2017	Minor methodological limitations (two studies with no limitations, one study with minor concerns, one with concerns on research design and data collection and one with moderate concerns on recruitment strategy)	Substantial concerns about relevance (all the studies included only Caucasian people)	Minor concerns about coherence (data reasonably consistent within and across all studies)	Minor concerns about adequacy (eight studies that offered together moderately rich data overall)	Moderate confidence	This finding was graded as moderate confidence because of minor concerns regarding methodological limitations, coherence and adequacy; though substantial concerns about relevance.

## 4. Discussion

This is the first meta-synthesis that focuses exclusively on the life experiences of people with migraine (either episodic or chronic). From our synthesis, four main themes were brought to the forefront: “Negative impact of migraine symptoms on overall life”; “Impact of migraine on family, work and social relationships”; “Impact of migraine on emotional health”; and “Coping strategies to deal with migraine”. Our findings are in line with the ones from the meta-synthesis of Nichols et al. on chronic headaches (Nichols et al., 2017). People with chronic headaches from different genesis share a detrimental experience akin to the participants of the studies in our review. This shared experience stemmed from a similar sense of suffering, difficulties in Organising work and household chores, blaming one's own situation and other psychological distress such as anxiety. Our themes can also overlap with the ones retrieved from two qualitative studies on adolescents with migraine (Donovan et al., 2013; Walter, 2017), which were excluded from this meta-synthesis as we focused only on adults. Nevertheless, the experience of overwhelming pain and a sense of isolation caused by migraine are present regardless the age. However, the need to share experiences and social support is more evident among adolescents than in our sample (Donovan et al., 2013; Walter, 2017).

The first theme, “Negative impact of migraine symptoms on overall life”, showed that migraine symptoms are disabling and affect everyday life. This is in line with the current quantitative literature about the quality of life of people with migraine (Blumenfeld et al., 2011; Haywood et al., 2018; Buse et al., 2019). These studies suggest that people with migraine experience high levels of disability that impact their health-related quality of life. The qualitative data from this meta-synthesis delve into the quantitative ones, explaining where the disability has its greatest impact. For example, Estave et al. explained how people with migraine experienced doing things without pleasure or wanting to do something, but their disease hindered this attempt (Estave et al., 2021).

However, the most significant burden of people with migraine emerges in the work and social fields, as we explained in the second theme, “Impact of migraine on family, work and social relationship”. This theme focused on how people with migraine perceived their disease to impact different spheres of life, namely, family, work and social relationships. When it comes to family and work, people with migraine reported these spheres to be hindered by migraine attacks. This is in line with a study by Buse et al., where the authors reported migraine harmed people's careers and the feeling of being “good parents” in one-third of their population (Stewart et al., 2010; Buse et al., 2019). Thus, quantitative data underlines the prevalence of negative impact on jobs, whereas qualitative data sheds some light on where these problems are. In particular, people with migraine reported the loss of cognitive function (concentration and memory) while at work due to their symptoms. This sense of discomfort is further worsened by the lack of understanding from their bosses. When it comes to intimate relationships, Buse et al. underlined the difficulty of people with migraine in establishing and maintaining a relationship, ending up breaking up with their partner because of the recurrence of attacks that affect the ability to do things together (Buse et al., 2019). Ruiz De Velasco et al. highlighted that migraine could also impact the sexual sphere because of the pain of migraine attacks and its negative consequences on sexual arousal (Ruiz De Velasco et al., 2003). Problems in sexual spheres for these people can be underrated by a general sense of embarrassment, stigma and cultural taboo. People during focus groups felt embarrassed to talk about this topic, while they felt more at ease during individual interviews. Talking about sex is a challenge in healthcare (Brandenburg and Bitzer, 2009). However, for some people, sexuality is an essential yet complex phenomenon to feel ashamed about. This aspect must be taken into account during the care process for people with migraine to offer them multidisciplinary support that tackles this disease from different perspectives.

The third theme, “Impact of migraine on emotional health,” underlines the effects of migraine on emotional health. In the studies retrieved in our meta-synthesis, people with migraine reported a general sense of guilt. One participant stated, “It's my brain. It's my fault” (Estave et al., 2021). This sense of guilt was reported by other participants, and it is an overarching theme that was recently pointed out as one of the elements that contribute to the migraine burden (Estave et al., 2021). Rutberg and Moloney highlighted that participants' guilt might also stem from the lack of awareness and understanding of this disease in society (Moloney et al., 2006; Rutberg and Öhrling, 2012). As regards the issue of not being understood by others, which could lead to isolation, Estave explained that improving knowledge and awareness of migraine in the general public could reduce emotional disorders in people with migraine (Estave et al., 2021). These burdensome feelings can be one of the reasons behind the high prevalence of psychological distress among people with migraine. To previous evidence, 23.1% of people with migraine experience psychological distress (Korkmaz et al., 2019; Donisi et al., 2020). The study by Chu et al. found that the severity of depression and anxiety are related to migraine frequency and can alter the perception of pain (Chu et al., 2018). Therefore, it is fundamental to consider also the psychological sphere when taking charge of people with migraine.

The final theme dealt with the “Coping Strategies to deal with migraine” that people with migraine brought to the forefront to deal with their disease. These strategies included the importance of self-efficacy, taking advantage of pain-free time, sharing experiences and balancing the demands of life. Palacios Ceña et al. underlined that their study participants wanted to go and live through the attacks, managing them (Palacios-Ceña et al., 2017). Believing in the ability to produce specific performance attainments in their available capacity is called “self-efficacy” (Gandolfi et al., 2019; Donisi et al., 2020). High levels of self-efficacy were reported as a key factor in preventing attacks and adaptation to pain (Gandolfi et al., 2019; Donisi et al., 2020). However, as written by Ramsey et al., they can push people to deal with pain and also to meet their and others' expectations, levering external motivation (Ramsey, 2012). The participants from our studies were aware of the importance of adopting different strategies to manage their disease. Some of them were more symptoms-related, like taking medications, going to a cold dark room to eliminate all external stimuli and resting as much as needed (Ramsey, 2012). Other strategies were more part of a more systemic management of the disease, such as sharing experiences to understand their conditions, and seeking social support from healthcare professionals, other people with migraine, friends, relatives and acquaintances (Belam et al., 2005). The benefit of this need is also confirmed by quantitative studies where higher perceived social support was positively correlated with lower migraine intensity and psychological distress (Gandolfi et al., 2019; Donisi et al., 2020). Moreover, pain-free time is essential to reduce triggers and control migraine attacks. Ramsey and Moloney explained that some of their participants used their pain-free time to do exercise and stress reduction activities (Moloney et al., 2006; Ramsey, 2012). Thus, multimodal management should be considered where these and other adaptative coping strategies are offered and shared with patients to handle their symptoms once there, increase their levels of self-efficacy and take the most out of their pain-free time.

Several limitations of this study need to be addressed. This meta-synthesis has a sample made mostly of Caucasian people. The participants in our meta-synthesis came mainly from America and Europe. Moreover, most of the participants were women. However, this is in line with the worldwide prevalence of migraine, which is more common in women than men. We included both episodic and chronic migraine, which could be limiting in understanding the perception of these two types of migraine. Nevertheless, the meta-synthesis by Nichols et al. (2017) on chronic headaches underlined similar themes. Finally, our studies drew their results upon different theoretical under-pinning, ranging from interpretative phenomenological analysis to grounded theory. This is a common challenge in the synthesis of qualitative research (Rahimi et al., 2009). In line with that, we tried to adopt different strategies to be as rigorous as possible. First, we created a well and focused research question. Then, we selected the studies following specific criteria deemed as meaningful to answer our research question. Moreover, our research team was composed of different professionals (e.g., physiotherapists and psychologists) to take into account the particular aspects of the primary studies. Finally, the primary studies highlighted a shared experience of the disease by people with migraine, no matter the adopted approaches. The strengths of these studies are the rigorous and sensitive research we performed with the help of a librarian and the fact that we included only participants with migraine diagnoses (ICHD criteria). Moreover, we use the CerQual to assess the certainty of the evidence of our findings.

## 5. Conclusions

This study synthetised the available evidence on the experience of people with migraine. Several spheres of quality of life are jeopardised, namely, work, social life, and sexual and emotional health. People with migraine felt to be unseen and stigmatised at work and during their social life as others struggle with understanding their condition. There is a need to tackle this disease from a social and health-policy point of view by educating people with migraine and those around them about this condition, making this disease more “visible” to society.

## Data availability statement

The original contributions presented in the study are included in the article/Supplementary material, further inquiries can be directed to the corresponding author.

## Author contributions

All authors listed have made a substantial, direct, and intellectual contribution to the work and approved it for publication.

## Acknowledgements

This work was developed within the DINOGMI Department of Excellence framework of MIUR 2018-2022 (Legge 232 del 2016). The authors would like to thank the librarians from Lund University for their assistance in creating the search string for this research.

## Footnotes

^1^The International Classification of Headache Disorders - ICHD-3. Available online at: https://ichd-3.org/ (accessed July 20, 2022).
