# Supplementary material for: Living with migraine: A meta-synthesis of qualitative studies
Source: Front Psychol. 2023 Mar 28;14:1129926. doi: 10.3389/fpsyg.2023.1129926 (PMC10086165; doi:10.3389/fpsyg.2023.1129926)
Supplement: Supplementary file 1 [file Table_1.DOCX]

Supplementary Material 1 – Search Strings

Living with Migraine: a Meta-Synthesis of Qualitative Studies

**Simone Battista, MSc^1,2^, Arianna Lazzaretti, BSc^1^, Ilaria Coppola, PhD^3^, Luca Falsiroli Maistrello, MSc^1,4^, Nadia Rania, PhD^3^, Marco Testa, PhD^1*^.**

1. Department of Neurosciences, Rehabilitation, Ophthalmology, Genetics, Maternal and Child Health, University of Genova, Campus of Savona, Italy

2. Department of Clinical Sciences Lund, Orthopaedics, Skåne University Hospital, Lund University, Lund, Sweden.

3. Department of Education Sciences, School of Social Sciences, University of Genova, Genova, Italy

4. Department of Physical Medicine and Rehabilitation, AULSS9 Scaligera, G. Fracastoro Hospital, 37074 San Bonifacio, Verona, Italy.

*** Correspondence:**Marco Testa, marco.testa@unige.it

**Search Strings**

**Medline via Pubmed:** ((("Migraine Disorders"[Mesh] OR "Headache Disorders, Primary"[Mesh] OR "Headache Disorders"[Mesh] OR Migraine OR Migrain* OR Headache OR Head pain) AND ("Surveys and Questionnaires"[Mesh] OR "Interview" [Publication Type] OR "Focus Groups"[Mesh] OR "Observation"[Mesh] OR “Nursing Methodology Research”[Mesh] OR "Hermeneutics"[Mesh] OR "Patient Acuity"[Mesh] OR "Grounded Theory"[Mesh] OR "Narration"[Mesh] Questionnaire OR Survey OR Interview OR Focus group OR Case stud* OR Observ* OR Qualitative research OR Qualitative method OR Hermeneutics OR Phenomenology OR Grounded theory OR narration OR Story-telling OR Storytelling OR Story telling)) AND ("Life Change Events"[Mesh] OR "Attitude"[Mesh] OR "Behavior"[Mesh] OR "Emotions"[Mesh] OR "Quality of Life"[Mesh] OR "Activities of Daily Living"[Mesh] OR "Social Participation"[Mesh] OR "Patient Participation"[Mesh] OR "Knowledge"[Mesh] OR "Health Knowledge, Attitudes, Practice"[Mesh] OR "Metacognition"[Mesh] OR "Perception"[Mesh] OR "Pain Perception"[Mesh] OR "Social Perception"[Mesh] OR "Self Concept"[Mesh] OR "Attitude"[Mesh] OR "Attitude to Health"[Mesh] OR "Emotions"[Mesh] OR "Behavior and Behavior Mechanisms"[Mesh] Experience* OR Opinion* OR Quality of life OR Belie* OR Feel* OR Attitude* OR Participation OR Emotional Involvement OR Self-concept OR Self concept OR Image OR View* OR perspective OR Perception OR Feeling* OR Behavi*)) AND ("Qualitative Research"[Mesh] Qualitative OR Mixed method) 🡪 422 entries 19/10/2021

**EMBASE:** ('migraine'/exp OR ('headache'/exp AND 'facial pain'/exp) OR 'headache'/exp OR migraine) AND ('questionnaire'/exp OR 'interview'/exp OR 'focus group'/exp OR 'focus group discussion'/exp OR 'focus group interview'/exp OR 'nursing methodology research'/exp OR 'hermeneutics'/exp OR 'observation'/exp OR 'patient acuity'/exp OR 'grounded theory'/exp OR 'phenomenology'/exp OR 'storytelling'/exp OR 'qualitative research'/exp OR 'survey'/exp) AND ('life event'/exp OR 'attitude'/exp OR 'behavior'/exp OR 'emotion'/exp OR 'quality of life'/exp OR 'daily life activity'/exp OR 'social participation'/exp OR 'patient participation'/exp OR 'knowledge'/exp OR 'attitude to health'/exp OR 'metacognition'/exp OR 'perception'/exp OR 'nociception'/exp OR 'self concept'/exp OR 'experience'/exp OR 'belief'/exp OR 'feeling'/exp OR 'participation'/exp OR 'image'/exp OR 'perspective'/exp OR opinion OR (emotional AND involvement)) AND ('qualitative research'/exp OR 'mixed method study'/exp OR 'mixed methods study'/exp OR 'mixed method'/exp OR 'mixed methods'/exp OR 'mixed methods research'/exp OR 'qualitative'/exp) AND [2000-2021]/py 🡪 272 entries 19/10/2021

**CINAHL, Psychinfo and Socindex:** ( migraine OR headache OR migraine headaches ) AND ( interview OR survey OR questionnaire OR focus group OR observation OR nursing methodology research OR hermeneutics OR patient acuity OR grounded theory OR narration OR phenomenology OR storytelling) AND (( life change events OR ( attitudes and behaviour ) OR emotions OR quality of life OR activities of daily living OR participation OR self concept OR health knowledge, attitudes, practice OR perception OR experience OR knowledge OR metacognition )) AND (qualitative OR mixed methods) 🡪 328 entries 19/10/2021

**Cochrane Library Central:** Search Name: Meta sintesi Migraine 2 75 Entries 19/10/2021

ID Search Hits

#1 MeSH descriptor: [Migraine Disorders] explode all trees 2812

#2 MeSH descriptor: [Headache Disorders] explode all trees 3541

#3 migraine 8447

#4 head pain 6109

#5 headache 35324

#6 #1 OR #2 OR #3 OR #4 OR #5 42848

#7 MeSH descriptor: [Surveys and Questionnaires] explode all trees 56906

#8 MeSH descriptor: [Interview] explode all trees 7

#9 interview 24445

#10 MeSH descriptor: [Observation] explode all trees 183

#11 MeSH descriptor: [Nursing Methodology Research] explode all trees 227

#12 MeSH descriptor: [Hermeneutics] explode all trees 2

#13 hermeneutic 31

#14 patient acuity 4192

#15 MeSH descriptor: [Grounded Theory] explode all trees 15

#16 MeSH descriptor: [Narration] explode all trees 212

#17 narration 282

#18 case study 66467

#19 story-telling 64

#20 story telling 101

#21 storytelling 285

#22 phenomenology 208

#23 grounded theory 670

#24 #7 OR #8 OR #9 OR #10 OR #11 OR #12 OR #13 OR #14 OR #15 OR #16 OR #17 OR #18 OR #19 OR #20 OR #21 OR #22 OR #23 145524

#25 MeSH descriptor: [Life Change Events] explode all trees 439

#26 life change events 12007

#27 MeSH descriptor: [Attitude] explode all trees 39430

#28 attitude 16297

#29 MeSH descriptor: [Behaviorism] explode all trees 2

#30 behavior 93570

#31 MeSH descriptor: [Emotions] explode all trees 18330

#32 emotion 7720

#33 MeSH descriptor: [Quality of Life] explode all trees 26469

#34 "quality of life" 125632

#35 MeSH descriptor: [Activities of Daily Living] explode all trees 9850

#36 "activities of daily living" 12002

#37 MeSH descriptor: [Patient Participation] explode all trees 1458

#38 participation 34853

#39 MeSH descriptor: [Health Knowledge, Attitudes, Practice] explode all trees 6180

#40 MeSH descriptor: [Metacognition] explode all trees 87

#41 metacognition 346

#42 MeSH descriptor: [Perception] explode all trees 18033

#43 perception 27590

#44 MeSH descriptor: [Self Concept] explode all trees 7386

#45 self concept 9640

#46 attitude 16297

#47 MeSH descriptor: [Attitude] explode all trees 39430

#48 experience 58534

#49 belief 3858

#50 believes 2081

#51 perspective 12317

#52 feeling 6576

#53 #25 OR #26 OR #27 OR #28 OR #29 OR #30 OR #31 OR #32 OR #33 OR #34 OR #35 OR #36 OR #37 OR #38 OR #39 OR #40 OR #41 OR #42 OR #43 OR #44 OR #45 OR #46 OR #47 OR #48 OR #49 OR #50 OR #51 OR #52 358714

#54 MeSH descriptor: [Qualitative Research] explode all trees 1197

#55 qualitative 18286

#56 mixed-method 829

#57 mixed method 11997

#58 #54 OR #55 OR #56 OR #57 28851

#59 #6 AND #24 AND #53 AND #58 1147
